# Hospital Note

**Published:** 1903-07

**Authors:** 


### HOSPITAL NOTE.

The Buffalo General Hospital will become the recipient of a legacy of $100,000 by the will of Mrs. Agnes Ethel Tracy, which was probated at New York, June 2, 1903.

By the fourth article Mrs. Tracy exercises a power of appointment given her under the will of her husband, Francis W. Tracy, respecting a fund of $100,000 in which Harriet Tracy, Mr.

Tracy's daughter by his first wife, has a life estate. This fund Mrs. Tracy directs be paid to the Buffalo General Hospital at the daughter's decease. She expresses the wish that it be in the nature of a memorial of her husband and his father, Albert H. Tracy, to perpetuate their memory in Buffalo.

